# The acute effects of a multi-ingredient pre-workout supplement on resting energy expenditure and exercise performance in recreationally active females

**DOI:** 10.1186/s12970-017-0206-7

**Published:** 2018-01-05

**Authors:** Michael Cameron, Clayton L. Camic, Scott Doberstein, Jacob L. Erickson, Andrew R. Jagim

**Affiliations:** 1 Exercise & Sport Science Department, University of Wisconsin – La Crosse, La Crosse, WI USA; 2 Department of Kinesiology and Physical Education, Northern Illinois University, DeKalb, IL 60115 USA; 3 Mayo Clinic Health Systems, Sports Medicine, Onalaska, WI 54650 USA; 4 Department of Exercise Science, Lindenwood University, St. Charles, MO 63301 USA

**Keywords:** Thermogenic, Ergogenic aid, Fatigue, Muscular endurance

## Abstract

**Background:**

The use of dietary supplements to improve performance is becoming increasingly popular among athletes and fitness enthusiasts. Unfortunately, there is a tremendous lack of research being done regarding female athletes and the use of sport supplements. The purpose of this study was to examine the acute effects of multi-ingredient pre-workout supplement (MIPS) ingestion on resting metabolism and exercise performance in recreationally-active females.

**Methods:**

Fifteen recreationally-active females participated in a randomized, double-blind, placebo controlled study. Subjects completed baseline, and two experimental testing sessions in a cross-over design fashion. Experimental testing included assessment of resting energy expenditure (REE), heart rate, and blood pressure following the ingestion of a MIPS or placebo. Subjects also completed a repetition to failure test for the back squat (BS) and bench press (BP) at 85% of their 5-repetition maximum followed by the assessment of anaerobic power using a counter-movement vertical jump test and a sprint test on a force-treadmill. Subjective measurements of energy, focus, and fatigue were also assessed using a 5-point Likert scale. Separate repeated measures analysis of variance (ANOVA) were used to assess differences in REE, cardiovascular responses, and subjective markers between conditions. Performance data were analyzed using paired Student’s T-tests.

**Results:**

A significant main effect for condition was observed for REE (*p* = 0.021) and diastolic blood pressure (*p* = 0.011) following ingestion of the MIPS. The supplement condition resulted in a greater number of BP repetitions to failure and total work completed during treadmill test (*p* = 0.039) compared to placebo (*p* = 0.037). A significant condition x time interaction for focus was observed with the supplement treatment exhibiting improved focus at 80-min post ingestion (*p* = 0.046).

**Conclusions:**

Consumption of a MIPS increased resting metabolism following a single dose accompanied by an increase in diastolic blood pressure. Furthermore, acute MIPS ingestion improved upper body muscular endurance and anaerobic capacity while improving feelings of focus following high-intensity exercise in recreationally active females.

## Background

The use of dietary supplements to enhance exercise performance and improve body composition has long been a popular strategy for active individuals [1]. A newer category of dietary supplements referred to as multi-ingredient pre-workout supplements (MIPS) have gained increased attention among active individuals. These products are a class of supplements that typically include a combination of ingredients such as caffeine, taurine, branch-chain amino acids, creatine, glutamine, and β-alanine [2, 3]. MIPS are often manufactured in a proprietary blend and designed to be ingested prior to a workout to enhance exercise performance and potentially lead to enhanced training adaptations over time. Based upon previous findings, the primary active ingredient in most pre-workout supplements appears to be caffeine as it is one of the few ingredients that offers fast-acting performance benefits [4–6], and when ingested alone is still a very popular ergogenic aid for athletes [7]. Caffeine levels peak in the body 30–60 min after ingestion and are widely distributed throughout all tissues [8] exerting its ergogenic effects in a variety of ways. More recent studies have focused on the effects of combining caffeine with a variety of ingredients, including beta-alanine, creatine, and various herbal extracts, for their potential synergistic benefits. Together, these ingredients offer specific physiological advantages, which often include enhanced energy availability, metabolism and improved buffering capacity of skeletal muscle [4, 5, 9].

Previous studies have reported improvements in acute muscular performance in individuals during upper and lower body exercises following ingestion of MIPS [2, 5, 9, 10]. For example, Beck et al. [9] observed a significant increase in upper body maximal strength in male subjects following acute ingestion of a caffeine-containing supplement. Similarly, Jagim et al. [2] reported a significant improvement in upper body muscular endurance performance following ingestion of a MIPS. In addition to these ergogenic benefits, MIPS, particularly those that contain caffeine, are also marketed as thermogenic agents due to their ability to increase metabolic activity and rates of lipolysis [11–13], potentially resulting in reductions in body weight over time. For example, Acheson et al. [11] observed an increase in metabolic rate after the consumption of caffeine in both individuals who were normal weight and obese. In a more recent study, Campbell et al. [12] reported increases in resting energy expenditure (REE) for up to 3-h in females who ingested a supplement containing caffeine and green tea extract. Long term, these acute increases in metabolic activity may lead to enhanced fat loss and improvements in body composition as a result of a higher total daily energy expenditure, particularly when combined with specific dietary or activity recommendations.

Manufacturers of MIPS often tend to make claims regarding their product’s ability to enhance energy levels and reduce sensations of fatigue during exercise. It is possible that if exercise is more enjoyable and less exhausting, individuals may participate longer, with greater intensity and more frequency; thereby potentially augmenting training adaptations over time. Several reports of benefits ranging from improvements in feelings of focus and energy to reductions in ratings of fatigue following consumptions of a MIPS during bouts of high-intensity exercise have been previously reported [2, 5, 14]. Specifically, Walsh et al. [15] reported improved ratings of focus, energy, and decreased feelings of fatigue, which also significantly improved time to exhaustion following consumption of a pre-workout supplement. Similarly, Jagim et al. [2] observed significantly increased subjective feelings of focus with a concomitant reduction in fatigue during a maximal effort sprint test following ingestion of a MIPS.

Although there have been multiple studies supporting the use of MIPS, the majority of the available research to this point has focused primarily on males. There are limited data regarding the effectiveness of pre-workout supplementation in female populations. Recently, a new product that was designed specifically for women has become available, with claims of increasing fat oxidation, increasing energy, and improving performance levels. The product has been shown to be safe for consumption in females [16], however, less is known regarding its influence on metabolism and performance. Therefore, the purpose of the current study was to examine the acute effects of ingesting a MIPS on REE, select clinical health markers, and exercise performance in recreationally active females. A secondary aim was to examine the effects of MIPS ingestion on subjective markers of focus, energy, and fatigue during exercise. It was hypothesized there would be an increase in REE, cardiovascular responses, and exercise performance following ingestion of the MIPS.

## Methods

### Overview

This study utilized a randomized, double-blind, placebo controlled cross-over design. Subjects first completed a familiarization session to become comfortable with the equipment and testing procedures prior to the experimental sessions. During the familiarization session, subjects completed a demographic form, a Physical Activity Readiness Questionnaire (PAR-Q), and exercise history form. Written consent was also obtained at this time in accordance with the Human Subject Guidelines and approved by the Institutional Review Board at the University of Wisconsin- La Crosse. Subjects reported to the human performance lab within 4–7 days of their familiarization session to complete baseline testing which included a body composition assessment, maximal strength testing, and further familiarization trials with the counter movement vertical jump test, and maximal sprint test. The participants were asked to fast for >2 h and abstain from exercise >24 h prior to baseline testing.

Within 7 days of baseline testing, subjects returned for the first of two experimental testing sessions. Subjects were again asked to fast for >8 h and abstain from vigorous exercise >24 h prior to experimental testing. Subjects first completed a questionnaire to assess their baseline feelings of focus, energy, and fatigue using a 5-point Likert scale (LS). Subjects then remained seated for a 3-min period followed by the assessment of resting heart rate (HR) and blood pressure (BP). Subjects then ingested either a placebo or the supplement in a randomized and double-blind fashion. Subjects were assessed for changes in REE, HR and BP over a 60-min period post-ingestion. At the end of 60 min, a second questionnaire was administered. Following the questionnaire, subjects completed a standardized dynamic warm-up lasting 10 min. Subjects then completed a counter movement vertical jump (CMVJ) test followed by muscular endurance testing using the bench press (BP) and back squat (BS) exercises. Following the muscular endurance testing, a third questionnaire was administered. Subjects rested for a 10-min period and then performed a 25-s maximal sprint test and completed the questionnaire for a fourth time. Four to seven days following the first testing session, subjects reported to the lab and completed the same protocol, receiving the opposite treatment. Subjects were encouraged to eat similar foods to what they ate prior to each testing session and completed a 2-day dietary history prior to each testing session, which was later assessed for total energy and macronutrient composition.

### Subjects

Fifteen recreationally active college-aged females were recruited to participate in this study (mean ± SD, age: 21.5 ± 1.7 y, height: 165.3 ± 5.3 cm, weight: 61.6 ± 5.1 kg, BF%: 22.9 ± 4.1%). Exclusion criteria included any contradictions to participation which included metabolic disorders, heart disease, arrhythmias, diabetes, thyroid disease, hypertension, hepatorenal, musculoskeletal, autoimmune, or neurological disease. Further exclusion criteria included taking prescription thyroid, cholesterol lowering, diabetic, anti-hypertensive, or anti-inflammatory medications. Inclusion criteria included being recreationally active when regular participation in aerobic and resistance training activities. Recreationally active was defined as participating in at least 150 min of moderate activity per week for at least six months based on the guidelines by American College of Sports Medicine [17] (mean ± SD, hours/week: 5.6 ± 2.2 h). Subjects were instructed to abstain from taking any nutritional supplements and/or ergogenic aids three weeks prior baseline testing, excluding a daily vitamin and/or protein supplementation. The participants were regular caffeine users with a mean caffeine intake value of 175 ± 26 mg/day. However, caffeine consumption was to be absent within one week of baseline testing until the final testing session.

### Testing procedures

#### Familiarization and baseline testing

During the familiarization session, subjects completed a practice trial for the CMVJ test and a maximal effort sprint test. Subjects then performed 10 repetitions of the BP and BS exercises to familiarize themselves with the equipment. Upon arrival to the laboratory for baseline testing, subjects first had their height and body mass determined according to standard procedures using a Healthometer scale (*Telstar LLC, Bridgeview, IL*). Body composition was then assessed using air displacement plethysmography (*BODPOD*, *Cosmed USA, Inc*.). Fat mass percentage values were determined based upon the body densities obtained from the BODPOD. Prior to each testing session, calibration procedures were completed according to the manufacturer guidelines using an empty chamber and a calibrating cylinder of a standard volume (49.55 L). Subjects were instructed to wear spandex or tight-fitting clothing, remove all jewelry, and wear a swim cap. Following body composition assessment, subjects completed a 10-min dynamic warm-up consisting of 4 min on a stationary cycle followed by 2 min of running on a standard treadmill at a speed of 6.0 mph and a set of dynamic stretches including both upper and lower body musculature. Following the warm-up, subjects completed a second CMVJ familiarization trial to get them further accustomed to the movement. Subjects then completed a 5-repetition maximum back squat (5RMBS) and 5-repetition maximum bench press (5RMBP) using an Optima Smith Machine (*LifeFitness, Schiller Park, IL*). Subjects initially completed a warm-up set of 5 repetitions at approximately 50% of their estimated 5RM. Next, subjects completed two sets of 5 repetitions at a load corresponding to 60–80% of their estimated 5RM with three minutes of rest in between. Subjects then performed subsequent sets of 5 repetitions of increasing weight to determine their 5RM. Three minutes of rest was provided in-between all attempts. All 5RM determinations were made within 1–3 attempts. A successful 5RMBS was determined by having the participant’s thighs parallel with the floor for each repetition. A successful 5RMBP was determined if the participant lowered the bar to their chest for each repetition. Subjects were encouraged to not pause at the top of each lift for more than a second for both 5RMBS and 5RMBP. Five minutes after maximal strength testing, the subjects practiced the maximal sprint test a second time which included two trials (15 s and the second 20 s).

### Experimental testing

#### Supplementation

Subjects were instructed to be fasted for 8 h and restrain from vigorous exercise >24 h prior to testing The MIPS tested in this study was a commercially available product supplied by the manufacturer (*MusclePharm, Fitmiss™ Ignite™).* The ingredients are listed in Table 1. The placebo (PLA) was matched for flavor and color and also provided by the manufacturer. Prior to testing, the assigned condition was prepared by an outside member of the research staff and delivered to the laboratory with the subject’s number on a shaker bottle in order to maintain a double blind procedure. Each powder was mixed in a shaker bottle with 16 oz. of cold water and a single-serving was ingested within 5 min.

**Table 1:** Supplement Ingredients

Serving Size: 1 Scoop (7.2 g)		
Amount Per Serving	Amount	% Daily Value
Calories	0	
Total Carbohydrates	1 g	<1%
Sugars	0 g	†
Calcium (as Calcium Silicate)	34 mg	3%
Proprietary FitMiss Ignite Blend	5700 mg	†

Other Ingredients:

Carnosyn® Patented Beta Alanine, Choline Bitartrate, L-Tyrosine, L-Glycine, Taurine, L-Carnitine Base, Beet Root Extract (Beta Vulgaris)(High in Nitrates), Hawthorn Berry Powder (Crataegus Pinnatifida)(Fruit), Agmatine Sulfate, Caffeine Anhydrous, Huperzine A 1% (*Huperzia Serrata*)

#### Resting measurements

Resting energy expenditure and respiratory exchange ratio were assessed using indirect calorimetry and with a TrueOne® 2400 metabolic measurement system (*ParvoMedics, Sandy, UT*). This test is a non-exertional test performed in a fasted state with the subjects lying supine on an exam Table. A clear, hard plastic hood and soft, clear plastic drape was placed over the subjects’ head and neck in order to determine resting oxygen uptake and energy expenditure. All subjects laid motionless without falling asleep for the duration of the 60-min period. The assessment of resting energy expenditure via indirect calorimetry using the TrueOne 2400 metabolic cart system has been shown to be a valid and reliable tool with within subject coefficient of variations ranging from 5.4–10.0% in male and female populations [18, 19]. Test to test reliability analysis of this model has yielded a mean intra-class coefficient (ICC) value of 0.942, *p* < 0.001 [19]. Heart rate and blood pressure were also assessed during this time using standard clinical procedures at time points of 15, 30, 45, and 60 min post ingestion.

#### Performance testing

Following REE assessment, subjects completed the same dynamic warm-up used during baseline testing. Subjects then completed a maximal effort CMVJ. Average power (W) and peak power (W) were later calculated using the participant’s body mass (kg), height (cm), and CMVJ height (cm) using previously described procedures [20]. Following the CMVJ test, subjects completed the muscular endurance testing using a resistance set at 85% of the pre-determined 5RM for the BS and BP respectively. Subjects were instructed to complete as many repetitions as possible during a single set to failure, while completing each repetition as “explosively” as possible without pausing at the top. Subjects were allowed a 5-min rest period between each exercise. Following a 10-min recovery period, the subjects performed a 25-s maximal sprint test on a non-motorized force treadmill (*Woodway, Waukesha, WI, USA*) set at a resistance of 12% of their bodyweight based upon previously used methods [21]. Subjects were given a 3-s count down and were instructed to sprint as fast as possible for the entire 25-s. Sprint tests were analyzed for total work completed, peak and average velocity and power.

#### Questionnaires

To assess feelings of focus, energy, and fatigue, a 5-point Likert scale was displayed to them. Specifically, subjects responded verbally to their rating on a scale of 1–5, which corresponded to: 1 = low, 2 = medium-low, 3 = medium, 4 = medium-high, 5 = high as has been previously used [2]. The questionnaire was administered at baseline, 60-min post-ingestion, post-bench press and immediately following the sprint test.

#### Dietary analysis

Each subject’s 2-day diet history was assessed using a commercially available nutrition analysis program (*MyFitnessPal, Inc.)* to assess differences in total energy and macronutrient intake during a 2-day period prior to each testing session to ensure the outcomes of the study were not influenced by dietary intake.

### Statistical analysis

All data were analyzed using the Statistical Package for the Social Sciences (SPSS Inc., Chicago, IL). Descriptive statistics were used to quantify baseline physical characteristics. Treatment (supplement or placebo) x time repeated measures analyses of variance (ANOVAs) were used to assess differences in REE (35, 60 min); Likert scale scores (0, 60, 80, 90 min) and HR and blood pressure (0, 15, 30, 45, 60, minutes). Paired student’s T-tests were used to assess differences in muscular endurance and anaerobic performance between each condition. Data were considered statistically significant when the probability of type I error was *p* < 0.05. If a significant interaction was observed for the ANOVA, a Tukey’s honest significant differences (HSD) post-hoc analysis was performed in order to determine where significance occurred between conditions.

## Results

### Resting energy expenditure

**Fig. 1:**
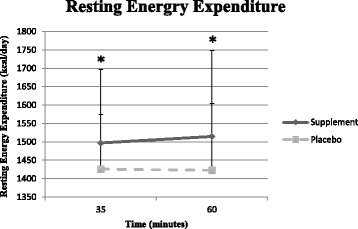
Changes in resting energy expenditure. *Significantly different between conditions (*p* < 0.05)

A significant main effect for condition was observed for REE (MIPS: 1497 ± 55.7; PLA: 1416 ± 42 kcal/day *p* = 0.043). Post-hoc analysis revealed the MIPS condition exhibited a higher REE at 35 min and 60 min post-ingestion as seen in Fig. 1. Due to machine complications, one participant’s data was discarded from the 35-min data time point.

### Hemodynamic variables

There were no significant differences observed for systolic blood pressure overall or between conditions following ingestion of the MIPS or PLA. There was a significant main effect for time (*p* = 0.002) observed regarding HR responses. Heart rate was significantly higher at baseline compared to each time point post-ingestion in both conditions. There was an overall main effect for condition regarding mean diastolic blood pressure following ingestion of the MIPS compared to the PLA (MIPS: 84 ± 1.1; PLA: 64.8 ± 1.3 mmHg, *p* = 0.011); however, no significant condition x time interaction (*p* = 0.44) was observed.

### Performance measures

**Fig. 2:**
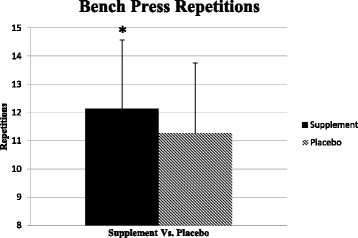
Mean bench press repetitions to failure. *Significantly different between conditions (*p* < 0.05)

Subjects completed a significantly higher number of BP repetitions to failure following ingestion of the MIPS condition compared to PLA (*p* = 0.037) as seen in Fig. 2.

There was no significant difference in BS performance between the MIPS and PLA (MIPS: 13.5 ± 3.7; PLA: 12.8 ± 3.1 reps, *p* = 0.28). Subjects completed a significantly greater amount of total work (m) during the 25-s treadmill sprint following ingestion of the MIPS compared to the PLA (MIPS: 93.97 ± 6.63; PLA: 93.01 ± 7.49 m, *p* = 0.039).

Table 2 provides a summary of CMVJ and treadmill performance variables following ingestion of the drinks.

**Table 2:** CMVJ and treadmill performance data (mean ± SD)

	MIPS (*n* = 15)	Placebo (n = 15)
Countermovement Vertical Jump		
Height (cm)	47.3 ± 3.9	47.3 ± 3.4
Average Power (W)	2012.8 ± 222.3	2016.3 ± 208.9
Peak Power (W)	3607.8 ± 451.9	3614.5 ± 409.7
Treadmill Performance		
Average Velocity (m/s)	3.7 ± 0.4	3.6 ± 0.3
Peak Velocity (m/s)	4.3 ± 0.5	4.2 ± 0.4
Average Power (W)	421.6 ± 61.1	419.8 ± 63.5
Peak Power (W)	1481.5 ± 235.7	1454.9 ± 331.2
Total Work (m) (*n* = 14)	94.0 ± 6.7*	93.01 ± 7.5

*Significantly different between conditions (*p* < 0.05)

### Dietary analysis

There were no significant differences between the subject’s mean energy and macronutrient intakes prior to each testing session as presented in Table 3.

**Table 3:** Summary of the 2-day dietary history for each condition

Measurement	MIPS (N = 15)	Placebo (N = 15)
Calories (kcals/d)	1597.7 ± 247.8	1638.1 ± 243.9
Carbohydrates (g)	219.2 ± 49.26	215.9 ± 208.9
Fat (g)	53.6 ± 20.9	55.8 ± 21.6
Protein (g)	69.7 ± 24.0	71.5 ± 22.9

Data are presented as Mean ± SD

### Side effects and subjective measures

**Fig. 3:**
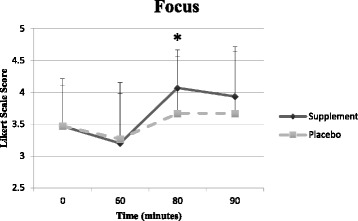
Changes in ratings of focus. *Significantly different between conditions (*p* < 0.05)

The side effects reported by the subjects included symptoms of having a *“flush face”, “tingly hands”*, and *“jitteriness”* (*n* = 4) approximately 30 min post-ingestion of the MIPS but not the PLA. A significant condition x time interaction was observed regarding feelings of focus. Post-hoc analysis revealed MIPS exhibited greater feelings of focus at 80 min post ingestion as seen in Fig. 3 (*p* = 0.046).

There was a main effect for time regarding feelings of energy throughout the workout for both conditions as subjects reported greater energy at 80 min post ingestion compared to energy levels at 90 min post ingestion (*p* < 0.01). A significant main effect for time regarding feelings of fatigue was observed for both conditions beginning 90 min post-ingestion (*p* < 0.001) however no significant condition x time interaction was observed (*p* > 0.05).

## Discussion

The purpose of the current investigation was to examine the acute effects of ingesting a commercially available multi-ingredient pre-workout supplement on REE, exercise performance and select markers of clinical health in recreationally active females. The primary findings from the current study indicated that ingestion of a MIPS resulted in an acute increase in REE. These results are in accordance with previous studies that have also observed significant increases in REE following ingestion of a MIPS or caffeine containing supplement [11, 12, 22]. For example, Outlaw et al. [22] reported significant increases in REE following ingestion of a commercially available caffeine-containing supplement (340 mg of caffeine) for 3 h post ingestion. Similarly, Campbell et al. [12] reported a significant increase in REE up to three hours post ingestion of a MIPS thermogenic supplement that contained 150 mg of caffeine plus green tea extract in healthy females. However, others have reported no significant changes in metabolic activity following ingestion of thermogenic agents. As evidenced, Rashti et al. [23] did not observe a significant increase in REE following ingestion of a caffeine-containing supplement. Although, it should be noted that the subjects from the Rashti et al. study [23] were only in a 3-h post absorption state suggesting that increases in REE may only occur during a period of an extended fast (>3 h). It is likely that the increases in REE observed in the current study is a result of the caffeine contained within the product as similar increases in REE have been shown to occur following consumption of caffeine alone [11]. Caffeine acts as an adenosine antagonist, which has a regulatory influence on metabolic activity. Therefore, when caffeine binds to adenosine receptors, it likely increase acute metabolic activity such as the case in the current study. This increase in REE is likely to persist even in those who are regular caffeine users as was also seen in the current study. This is further supported by the aforementioned study done by Outlaw et al. [22] who observed the significant increase in REE at 60 min post-ingestion of a caffeine-containing supplement, even though the subjects were regular caffeine consumers and reported average caffeine intakes of approximately 200 mg per day.

Furthermore, results from the current study also indicate that MIPS ingestion does not appear to negatively influence HR in recreationally active females as HR remained stable and did not significantly increase following consumption of a MIPS compared to a PLA. This finding is consistent with other studies [3, 12] which also observed minimal changes in HR following ingestion of a MIPS. Conversely, some studies [24, 25] have reported increases in HR following consumption of a MIPS, which may be attributable to differences in the amount of caffeine consumed or the caffeine habits of the subjects.

Based upon the results of the current study, it also does not appear as though systolic blood pressure is significantly influenced by MIPS ingestion. These findings are in contrast to previous studies, which have observed significant increases in systolic blood pressure following consumption of MIPS or caffeine-containing beverages [3, 12, 25]. For example, Campbell et al. [12] reported a significant increase in both systolic and diastolic blood pressure following ingestion of a caffeine-containing thermogenic supplement in female subjects. Interestingly, a significant increase in only diastolic BP was observed in the current study. These contradictory findings may again be attributable to the differences in caffeine dosage. As stated previously, caffeine is an adenosine antagonist and therefore acts as a vasoconstrictor. This vasoconstriction can lead to an increase in systemic vascular resistance and the isolated rise seen in diastolic pressure in this study may be explained by this as vascular resistance impacts diastolic blood pressure to a greater degree than systolic pressure. A change in diastolic blood pressure of this magnitude however is rather abnormal, particularly under resting conditions and warrants further investigation. For the current investigation, the MIPS contained a blend of caffeine and various other ingredients, however, the exact amounts are unknown. Previous studies have observed changes in HR and systolic BP following ingestion of caffeine-containing beverages with dosages ranging from 230 to 495 mg, which may explain some of the inconsistencies regarding hemodynamic responses [3, 12, 24, 25]. Further, acute increases in diastolic blood pressure may be a concern for those with cardiovascular disorders. However, a recent study by Vogel et al. [5] investigated the same MIPS used in the current study and determined it to be safe for females to consume 1 or 2 servings of a MIPS daily for 28 days as evidenced by a lack of change in hematological markers or resting vitals in recreationally active females. Based on the results of the current study and those from Vogel et al. [5], it appears as though the supplement does not result in any serious adverse effects with the exception for the potential rise in diastolic blood pressure.

The current investigation observed a significant increase in upper body muscular endurance, which is in agreement with the previous findings that have investigated the acute effects of MIPS on various aspects of performance [2, 26, 27]. It can be assumed that the caffeine contained within the current MIPS likely played a significant role in the observed performance benefits, as it is one of the few ingredients with an immediate mechanism of action as described earlier and has been previously shown to improve performance [6]. It has been proposed that caffeine may positively influence muscular endurance by its direct effect on muscle anaerobic energy provision and its ability to increase muscle contractility [26]. Further, caffeine also acts as a central nervous stimulant and therefore may delay the onset of fatigue or allow individuals to better tolerate a higher training intensity ultimately allowing for a greater work capacity [24, 26]. A recent meta-analysis [6] found that caffeine ingestion appears to improve muscular endurance (overall ES = 0.28, *p* < 0.01) and increase maximum voluntary contraction, particularly during lower body exercises (overall ES = 0.67, *p* < 0.01). However, the current investigation did not observe a significant increase in lower body muscular endurance. These findings are in opposition to a prior investigation, which observed a significant increase in lower body endurance, but not upper body [24] following ingestion of a MIPS in males. These differences in performance outcomes may be explained by differences in instrumentation utilized (i.e., leg press versus the back squat) or too low of a caffeine amount to elicit a positive improvement in lower body performance. It is worth noting that other ingredients in the MIPS may also have contributed to the improvement in performance as some of them are designed to also enhance exercise performance. For example, tyrosine supplementation is purported to reduce sensations of central fatigue during strenuous exercise however the supporting evidence is not clear as previous studies have failed to detect any substantial improvements in strength, exercise capacity or anaerobic power [27, 28]. Beet-root extract is also purported to enhance exercise performance by augmenting the vasodialatory response to exercise thereby improving blood flow. There is some evidence to support its ability to improve exercise performance however, as a result of the proposed mechanism of action, it is not likely to influence the exercise modalities used in the current study in addition to the fact that prior research also suggests that multiple doses overall several days may be required in order to elicit an ergogenic benefit [29–31]. Additionally, it would be expected that if adequate amounts of boot root extract were present, reductions in blood pressure would have occurred as a result of increased vasodilation and antihypertensive responses which is contradictory to the present findings. Similarly, beta-alanine supplementation has also been shown to enhance exercise performance, particularly during bouts of high-intensity exercise lasting 30–180 s. as a result of an improvement in buffering capacity. However, it is again believed that several dosages over the course of multiple weeks (~4 weeks) is required to substantiate any performance benefits [32]. Additionally, the dosage of beta-alanine required for an ergogenic benefit appears to be 4–6 g/d [32] and the MIPS used in the current study only contains 5.6 g of a proprietary blend which also contains several other ingredients. Therefore, the likelihood of there being enough beta-alanine present to elicit any acute benefit is low. Taurine is another ingredient that has been proposed to enhance anaerobic performance [33]; however, once again, a larger dose (~5 g) is likely required to substantiate any ergogenic benefit.

In the current investigation ingestion of the MIPS did not appear to influence lower body power, which is in accordance with previous findings. For example, Jagim et al. [2], also did not observe a significant improvement in lower body power following ingestion of a MIPS. However, Jagim et al. [2] did observe a significant increase in mean power during a maximal effort sprint test. Adversely, the current investigation observed a significant increase in total work during the maximal sprint following ingestion of the MIPS but not power. All other anaerobic power measurements during the maximal sprint test were not significantly different following ingestion of a MIPS compared to the PLA, which is similar to results seen in previous studies [2, 8].

A secondary aim of the current study was to examine how MIPS ingestion influences subjective markers of focus, energy, and fatigue during exercise. Based upon the results of the current investigation, it appears as though acute MIPS ingestion may positively influence feelings of focus following a bout of high-intensity exercise. The findings of the current investigation are in agreement with the observations from a previous study [15] during which subjects reported statistically greater feelings of focus and energy 10-min into running on a treadmill at 70% of $\dot{\mathrm{V}}$O_2_max following ingestion of a MIPS. However, the current study did not observe a statistical difference in subjective feelings of energy or fatigue levels post exercise following ingestion of a MIPS compared to PLA. These findings are in opposition of Jagim et al. [2] who reported reductions of fatigue levels throughout testing following ingestion of a MIPS during a strength training protocol. This disparity between the two conditions may be more drastic due to the greater workload of a strength training protocol consisting of 5 sets of 5 repetitions with a 6th set to failure [2]. Additionally, differences in ingredient amounts, particularly caffeine, across different MIPS’s may also yield contradictory findings relating to subjective measures of fatigue and energy levels. It is likely that caffeine contained within the MIPS was primarily responsible for the observed increase in focus as previous research has observed similar findings during high-intensity exercise following consumption of caffeine-containing beverages [34, 35]. Tyrosine, which is also one of the included ingredients, has also been proposed to improve cognitive performance during exercise [36]. For example, Deijen et al. [36] observed improvements in cognitive performance during periods of physical stress in military cadets however a relatively large dose was used in the study and the supplement was ingested for a 6-day period.

## Conclusions

Ingestion of a MIPS appears to result in acute elevations in REE up to 60 min post ingestion in recreationally active females. These elevations appear to occur with minimal changes in heart rate and systolic blood pressure however an increase in diastolic blood pressure was observed and individuals with risk factors for cardiovascular disease may need to exercise caution prior to consuming such a product. Further, the current MIPS appears to positively influence upper body muscular endurance, which could enhance training adaptations over time by allowing for a greater training volume. The added improvement of the subjective measure of focus could further improve the quality of a training session. Additional research is needed to examine the long-term effects of MIPS ingestion on measures of clinical health and training adaptations in female populations.
